# Detection of SARS-CoV-2 antibodies is insufficient for the diagnosis of active or cured COVID-19

**DOI:** 10.1038/s41598-020-76914-5

**Published:** 2020-11-16

**Authors:** Pilar Escribano, Ana Álvarez-Uría, Roberto Alonso, Pilar Catalán, Luis Alcalá, Patricia Muñoz, Jesús Guinea

**Affiliations:** 1 Clinical Microbiology and Infectious Diseases, Hospital General Universitario Gregorio Marañón, Madrid, Spain; 2 Instituto de Investigación Sanitaria Gregorio Marañón, Madrid, Spain; 3 Medicine Department, Faculty of Medicine, Universidad Complutense de Madrid, Madrid, Spain; 4 CIBER Enfermedades Respiratorias-CIBERES (CB06/06/0058), Madrid, Spain

**Keywords:** Diagnostic markers, Infectious-disease diagnostics

## Abstract

We assessed the performance of Abbott's SARS-CoV-2 IgG assay and the Panbio^TM^ COVID-19 IgG/IgM rapid test device for the diagnosis of either active or cured COVID-19. Three cohorts of patients were chosen. Cohort 1, patients (n = 65) who attended the emergency department on March 30, 2020 with clinical suspicion of active COVID-19 (n = 56 with proven/probable COVID-19). Cohort 2, hospital workers (n = 92) who had either been (n = 40) or not (n = 52) diagnosed with proven/probable COVID-19 and were asymptomatic at the time of the sampling. Cohort 3, patients (n = 38) cared at the hospital before the start of the COVID-19 pandemic. Detection of serum antibodies was done using Abbott´s SARS-CoV-2 IgG assay and the Panbio^TM^ COVID-19 IgG/IgM device. Both methods showed 98% agreement for IgG detection. No antibodies were detected in the 38 samples from hospitalized pre-COVID subjects. The diagnostic performance of IgGs detected by Abbott´s SARS-CoV-2 assay in Cohorts 1/2 was: sensitivity (60.7%/75%) and specificity (100%/84.6%). The diagnostic performance of IgM by Panbio^TM^ COVID-19 in Cohorts 1/2 was: sensitivity (16%/17.5%) and specificity (100%/98.1%). We show that IgG detection alone is insufficient for the diagnosis of active or cured COVID-19. IgM detection has a limited diagnostic value.

## Introduction

The COVID-19 epidemic, which started in late December 2019 in China, rapidly spread to many countries affecting millions of people worldwide^1,2^. The clinical presentation may range from asymptomatic or mild illness to severe pneumonia, acute respiratory distress syndrome, and progress to severe and fatal respiratory failure and death. Symptoms usually emerge 2–14 days after viral exposure and COVID-19-positive patients may experience fever, cough, fatigue, muscle pain, and shortness of breath, among the most commonly reported clinical manifestations^3,4^.

To date, the diagnosis of COVID-19 is mainly based on the detection of SARS-CoV-2 virus RNA by PCR from samples of patients under clinical suspicion. However, PCR sensitivity is 80% at best, with lower values in early and late stages of the infection^5^. Furthermore, and given the large number of infected patients who probably have not been tested or reported as having PCR false-negative results, detection of viral RNA is insufficient to assess the current number of people affected by COVID-19^6^.

The detection of IgG antibodies against SARS-CoV-2 is possible in serum samples of patients with active COVID-19 in the first week after the onset of the infection^7^. This potentially allows to improve the microbiological diagnosis, along with RNA detection from nasopharyngeal swabs, in active cases and also to detect cured cases^8^. The serological detection of IgG against SARS-CoV-2, mostly using in-house procedures, has shown a sensitivity that ranges between 78 and 100% in patients requiring hospital admission^7–15^. Abbott´s SARS-CoV-2 IgG assay is a recent commercial chemiluminescence immunoassay (CLIA) platform approved by the FDA that allow detecting IgG in serum samples and has been validated in samples from hospitalized patients with COVID-19^16^. On the other hand, the Panbio^TM^ COVID-19 IgG/IgM rapid test is a point-of-care lateral flow device that may be a good alternative to CLIA-based procedures. However, the role of IgG detection for the diagnosis of cured COVID-19 remains an enigma, and, to the best of our knowledge a comparison of the two diagnostic platforms has not been done yet.

Here compared Abbott's SARS-CoV-2 IgG assay and the Panbio^TM^ COVID-19 IgG/IgM for IgG detection. We also assessed the diagnostic performance of the IgG and IgM detected for the diagnosis of active and cured COVID-19.

## Results

### Description of study patients

Cohort 1 included 65 patients with clinical suspicion of active COVID-19 who were cared at the hospital´s emergency department. Table 1 shows the clinical characteristics of these patients. Sixty per cent were male and median age of 56 years. At the time of the sampling, all patients had signs and symptoms compatible with COVID-19, most with (81.5%) pneumonia. Fever (84.6%), cough (81.5%), and dyspnoea (61.5%) were the most commonly observed symptoms. Chest X-rays showed lung opacities in 53 (81.5%) of the cases. Fifty-six patients had either proven (n = 49) or probable COVID-19 (n = 7), whereas the remaining nine patients were categorized as not infected.

**Table 1:** Clinical characteristics of subjects suspected of having active COVID-19 (Cohort 1) and comparisons between patients with detectable and undetectable IgGs.

	Cohort 1			Subjects with proven/probable COVID-19	
	Overall	Proven/Probable COVID-19	COVID-19 free	IgG-positive	IgG-negative
Median age, in years (IQ range)	56 (45.5–68)	56 (46.25–67.8)	50 (35.5–70.5)	60 (47–69.5)	53.5 (44.75–63.5)
Male	36 (60%)	36 (64.3%)	3 (33.3%)	22 (64.7%)	14 (63.6%)
Presence of symptoms	100%	100%	100%	ND	ND
Days from onset of symptoms to serum sampling (IQ range)	7 (4.25–10)	7 (5–10)	5 (3–6.5)	7 (5–13.25)	7 (4–10)
Fever (> 38 °C)	55 (84.6%)	49 (87.5%)	6 (66.7%)	**27 (79.4%)**	**22 (100%)**
Headache	11 (16.9%)	11 (19.6%)	9 (100%)	5 (14.7%)	6 (27.3%)
Cough	53 (81.5%)	48 (87.5)	5 (55.6%)	30 (88.2%)	18 (81.8%)
Asthenia	32 (49.2%)	29 (51.8%)	3 (33.1%)	17 (50%)	12 (54.5%)
Myalgia	19 (29.2%)	18 (32.1%)	1 (11.1%)	9 (26.5%)	9 (40.9%)
Sore throat	2 (3.1%)	1 (1.8%)	1 (11.1%)	0 (0%)	1 (4.5%)
Runny nose	3 (4.6%)	1 (1.8%)	2 (22.2%)	1 (2.9%)	0 (0%)
Dyspnoea	40 (61.5%)	34 (60.7)	6 (66.7%)	24 (70.6%)	10 (45.5%)
Pneumonia	53 (81.5%)	53 (94.6%)	0 (0%)	**34 (100%)**	**19 (86.4)**
Anosmia	7 (10.8%)	6 (10.7%)	1 (11.1%)	2 (5.9%)	4 (18.2%)
Ageusia	6 (9.2%)	5 (8.9%)	1 (11.1%)	1 (2.9%)	4 (18.2%)
Abdominal pain	4 (6.2%)	1 (1.8%)	3 (33.3%)	0 (0%)	1 (4.5%)
Diarrhoea	24 (36.9)	20 (30.7%)	4 (44.4%)	10 (29.4%)	10 (45.5%)
Hospital admission	45 (59.2%)	42 (75%)	3 (33.3%)	27 (79.4%)	15 (68.2%)
Death	6 (9.2%)	5 (8.9%)	1 (11.1%)	2 (5.9%)	3 (13.6%)
Positive IgG	34 (52.3%)	34 (60.7%)	0 (0%)	NA	NA
Number of patients	65	56	9	34	22

ND, not done; NA, not applicable. Numbers in bold indicate differences reaching statistical significance (*P* < 0.05).

Cohort 2 was made up by 92 hospital workers with criteria of proven (n = 39) or probable cured COVID-19 (n = 1), or asymptomatic (n = 52). Table 2 summarizes the characteristics of study patients with proven/probable cured COVID-19; 40% were male with median age of 49 years. All patients had had symptoms compatible with COVID-19 and 13 (32.5%) had had pneumonia in the previous month. Headache (77.5%), cough (57.5%), asthenia (62.5%), and myalgia (67.5%) were the most commonly observed symptoms.

**Table 2:** Clinical characteristics of subjects with proven/probable cured COVID-19 (Cohort 2) and comparisons between patients with detectable and undetectable IgGs; NA, not applicable.

	Patients with proven/probable cured COVID-19		
	Overall	IgG-positive patients	IgG-negative patients
Median age, in years (IQ range)	49 (36.25–55)	50 (36.7–55.2)	40 (34–55.5)
Male	16 (40%)	13 (43.3%)	3 (30%)
Presence of symptoms	40 (100%)	30 (100%)	10 (100%)
Days from onset of symptoms to serum sampling (IQ range)	26 (22–32)	26 (22.5–31.5)	26.5 (19.5–32.5)
Fever (> 38 °C)	19 (47.5%)	16 (53.3%)	3 (30%)
Headache	31 (77.5)	21 (70%)	10 (100%)
Cough	23 (57.5)	19 (63.3%)	4 (40%)
Asthenia	25 (62.5)	19 (63.3%)	6 (60%)
Myalgia	30 (75%)	22 (73.3%)	8 (80%)
Sore throat	11 (27.5%)	20 (33.3%)	1 (10%)
Runny nose	13 (32.5%)	12 (40%)	1 (10%)
Dyspnoea	16 (40%)	12 (40%)	4 (40%)
Pneumonia	13 (32.5%)	10 (33.3%)	3 (30%)*
Anosmia	18 (45%)	**17 (56.7%)**	**1 (10%)**
Ageusia	17 (42.5%)	15 (50%)	2 (20%)
Abdominal pain	5 (12.5%)	5 (16.7%)	0 (0%)
Diarrhoea	10 (25%)	8 (26.7%)	2 (20%)
Hospital admission	1 (2.5%)	1 (3.3)	0 (0%)
Death	0 (0%)	0 (0%)	0 (0%)
Positive IgG determination	30 (75%)	NA	NA
No. of patients	40	30	10

Numbers in bold indicate differences reaching statistical significance (P < 0.05). *Chest X-rays were performed in 22 out of the 40 patients.

Statistically significant differences were determined when comparing patients with proven/probable COVID-19 from Cohorts 1 and 2. Patients from Cohort 1 were older, the number of days between the onset of symptoms to serum sample collection were lower, and showed higher frequency of fever, cough, dyspnoea, pneumonia, and hospital admission. The most frequent symptoms in patients from Cohort 2 were headache, myalgia, sore throat, rhinorrhoea, anosmia, ageusia, and abdominal pain.

### Performance of Abbott´s SARS-CoV-2 IgG assay for the diagnosis of COVID-19

From the 38 samples from patients with either community-acquired pneumonia of unknown etiology or coronavirus infection other than SARS-CoV-2, none generated a positive result (index value below 0.2). IgG cross-reactivity with patients without COVID-19 was thus ruled out.

In Cohort 1, IgGs were detectable in samples from 34/65 patients; sensitivity, specificity, positive predictive value, and negative predictive value of IgG detection for the diagnosis of active COVID-19 were 60.7%, 100%, 100%, and 29.3%, respectively (Table 3). Serum samples from patients with proven/probable COVID-19 were collected at a median of seven days (IQR 5–10 days) from the onset of symptoms. There was no improvement in the sensitivity in serum samples taken from patients reporting disease onset > 7 days, nor by excluding probable cases. Twenty-two COVID-19-positive patients – most of which were proven cases (n = 20) – showed negative IgGs. We further compared patients with proven/probable COVID-19 with detectable or undetectable IgGs and found that patients with detectable IgGs had higher frequency of > 38 °C fever and/or pneumonia (P < 0.05). The remaining comparisons resulted in differences without statistical significance, although a trend towards higher frequency of dyspnoea in IgG-positive patients (P = 0.055; Table 1) was detected. Sensitivity of IgG detection was higher in patients with probable COVID-19 than in subjects with proven COVID-19 and it was unrelated to the number of days from the onset of symptoms. All patients not fulfilling COVID-19 criteria had negative serum IgGs.

**Table 3:** Sensitivity, specificity, positive predictive value, and negative predictive value of IgG detection in serum samples for the diagnosis of active and cured COVID-19. PPV, positive predictive value; NPV, negative predictive value; NA, not applicable.

	IgG determination		Diagnostic performance			
	Positive	Negative	Sensitivity	Specificity	PPV	NPV
**Cohort 1**						
*Proven and probable COVID-19 (n* = *56)*						
≤ 7 from onset of symptoms	19	11	63.3	100	100	42.1
> 7 from onset of symptoms	15	11	57.7	100	100	8.3
Overall	34	22	60.7	100	100	29.3
*Proven COVID-19 (n* = *49)*						
≤ 7 from onset of symptoms	16	10	61.5	100	100	44.4
> from onset of symptoms	13	10	56.5	100	100	9.1
Overall	29	20	59.2	100	100	31.1
COVID-19 free (n = 9)	0	9	NA	NA	NA	NA
**Cohort 2**						
*Proven and probable cured COVID-19 (n* = *40)*						
Overall	30	10	75	84.6	79	81.5
*Proven cured COVID-19 (n* = *39)*						
Overall	29	10	74.4	84.6	78.4	81.5
COVID-19 free (n = 52)	8	44	NA	NA	NA	NA

In Cohort 2, IgGs were detectable in samples from 38/92 patients; sensitivity, specificity, positive predictive value, and negative predictive value of IgG detection for the diagnosis of cured COVID-19 (Cohort 2) were 75%, 84.6%, 79%, and 81.5%, respectively (Table 3). Median number of days for sample collection from the onset of symptoms in patients with proven/probable cured COVID-19 was 26 days (IQR 22–32 days). Ten patients with proven cured COVID-19 resulted in negative IgG and serum samples of 8 of the patients, taken one month later, remained negative. Anosmia was more frequent in patients with proven/probable cured COVID-19 and detectable IgGs than in subjects without detectable IgGs (P < 0.05) (Table 2). No statistically significant differences were observed for the remaining comparisons. We detected IgGs (index values ranging between 2.08 and 6.75) in serum samples from eight patients not fulfilling COVID-19 criteria; four of them reported mild symptoms in the two months before sample collection: headache or anosmia (n = 3); cough, dyspnoea or ageusia (n = 2); myalgia, sore throat or rhinorrhoea (n = 1 each). Two women receiving prophylaxis with fosamprenavir were asymptomatic carriers who resulted PCR-positive and IgG-negative while participating in the study; one of them developed symptoms few days after the end of the study, suggesting an early detection COVID-19 case.

**Figure 1:**
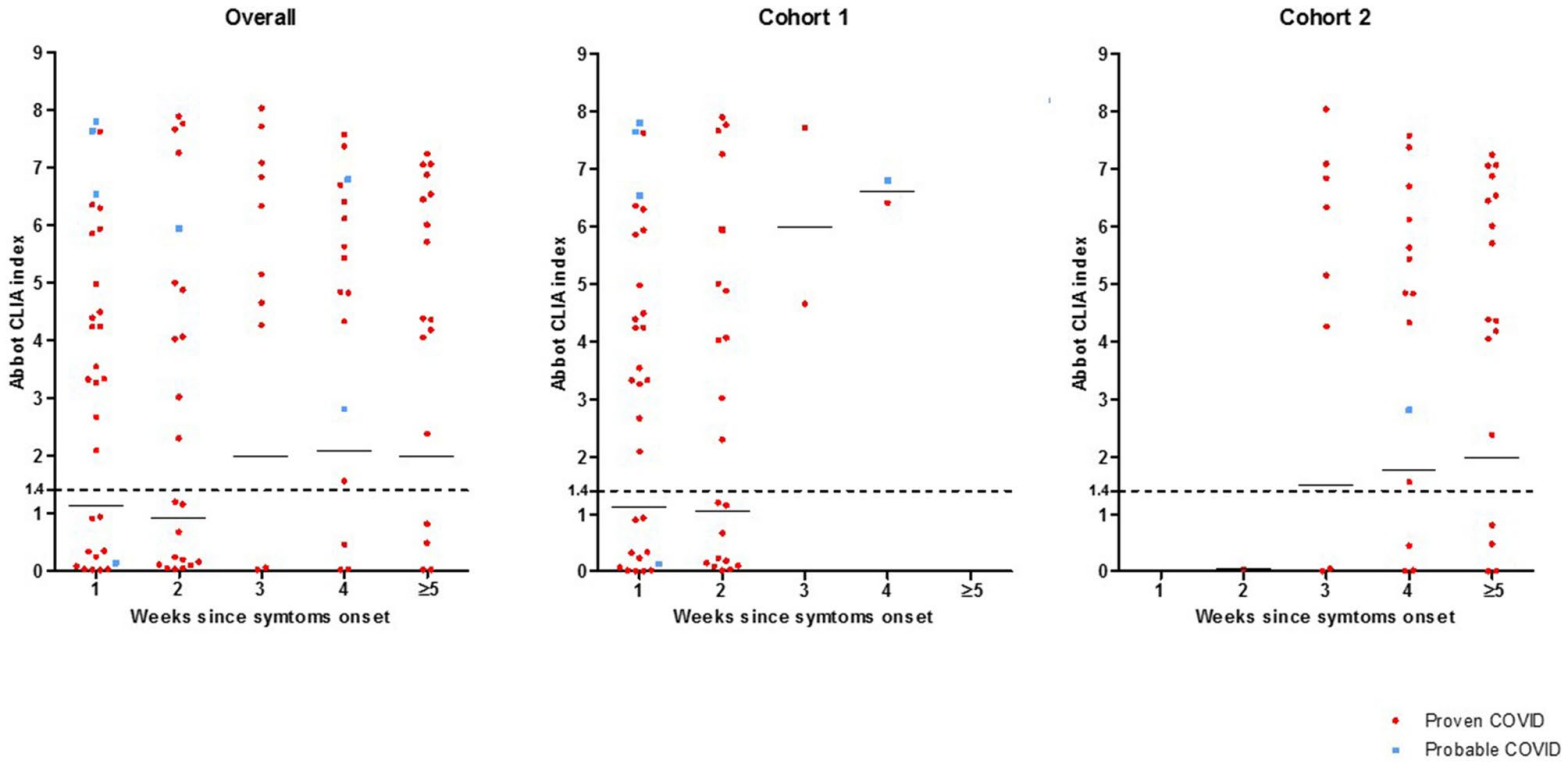
Scatter plots of index values obtained by Abbott´s SARS-CoV-2 IgG assay (Architect analyser) versus days from the onset of symptoms of all patients (**a**), and patients from Cohort 1 (**b**) and Cohort 2 (**c**). Dots in red indicate proven COVID-19 cases and dots in blue indicate probable COVID-19 cases.

Index values of Abbott´s SARS-CoV-2 IgG assay are shown in Fig. 1. A number of patients in Cohort 1 who reported onset of symptoms in the previous seven days had IgG-positive results, some of them showing high index values. Patients in Cohort 2 showed incremental index values over time, but IgG-negative cases commonly showed undetectable index values. Overall, seven out of the eight patients with probable COVID-19 had high IgG-positive index values (> 2.5).

### Performance of the Panbio^TM^ COVID-19 IgG/IgM rapid test device for the diagnosis of COVID-19 comparisons with Abbott´s SARS-CoV-2 IgG assay

No antibodies were detected in the 38 samples from patients cared at the hospital in the period before the COVID-19 epidemic. The Panbio^TM^ COVID-19 IgG/IgM rapid test device showed absence of antibodies in 85 samples, presence of IgMs and IgGs in 20 samples, and presence of either IgM alone (n = 3) or IgG alone (n = 49) in the remaining samples. IgM antibodies were detected in 15 patients from Cohort 1 (26% and 100% sensitivity and specificity, respectively) and in eight patients from Cohort 2 (17.5% and 98.1% sensitivity and specificity, respectively). The three patients with only positive IgM determinations had proven COVID-19 (n = 2) or were not infected (n = 1). Based on the presence of IgM, IgG, or both, the sensitivity and specificity of the Panbio^TM^ COVID-19 IgG/IgM rapid test device for the diagnosis of COVID-19 in patients from Cohort 1 and Cohort 2 were 60.7% and 100%, and 77.5% and 86.5%, respectively.

IgG detection in the 157 serum samples tested using both procedures showed high concordance: positive (n = 69) and negative (n = 85); three samples (1.9%) yielded discrepant positive results with Abbott´s SARS-CoV-2 IgG or negative results by the Panbio^TM^ COVID-19 IgG/IgM rapid test device. Discrepancies were retested (CLIA index results ranging between 2.1 and 3.3), resulting in two patients from Cohort 2 who were not infected and one patient with proven COVID-19 from Cohort 1.

## Discussion

Our study shows that serum IgG detection alone is insufficient for the diagnosis of active or cured COVID-19, with sensitivity values that range between 60 and 75%, respectively. Detection of IgM adds limited value to the performance of serological strategies. However, the coupled detection of SARS-CoV-2 RNA and IgG may improve the performance of any of the two procedures alone.

As PCR is insufficient for the diagnosis of active COVID-19, alternative procedures are essential. Non-PCR based procedures rely on the detection of either viral antigens or antibodies against SARS-CoV-2^17^. Antigen detection has shown sensitivity and specificity values ranging between 50 and 100%, respectively. Low viral loads and variability in a sample collection limit sensitivity^18^. IgG detection may improve and speed up the diagnosis of COVID-19 in symptomatic patients, particularly in negative SARS-CoV-2 PCR cases. It has been shown that in up to 15% of COVID-19-positive patients IgG antibodies are detectable during the first week following the onset of symptoms^8^, although reaching 100% of the patients required two additional weeks^8,12,13,19^. Here, we examined the performance of serology as an adjuvant for the diagnosis of COVID-19 using commercially available procedures. Detection of IgG antibodies using Abbott´s SARS-CoV-2 IgG assay (Architect analyser) and the Panbio^TM^ COVID-19 IgG/IgM rapid test device resulted in high agreement (98%). To the best of our knowledge, to date there is only one study that has assessed Abbott´s SARS-CoV-2 IgG assay (Architect analyser) in samples from patients admitted to the hospital^16^. The performance of the test was particularly good in samples collected ≥ 10 days from the onset of the disease. Bryan et al. report 100% sensitivity in cases for which samples were taken ≥ 17 days from the onset of the disease. The results communicated by these authors are significantly higher than the ones reported in our study^16^. Here, we tested patients who attended the emergency department with acute and active clinical presentations (Cohort 1). Our serum samples were collected at a median of seven days from the onset of symptoms, mostly < 10 days. This may explain the high number of IgG-negative determinations in patients with proven COVID-19. Of note, there is a trend of positive samples that show high index values (> 2), particularly in probable COVID-19 cases (Fig. 1). Coupling IgG detection to PCR testing may have helped increase the number of COVID-19 cases from 49 (proven cases) to 56 (proven and probable cases).

Another added value of IgG detection is to unearth undetected and cured COVID-19 cases in order to assess the proportion of the population that is protected (serological status) and understand the real burden of the disease. At this point, serology may be the only method to track down cured infected patients. Here, we confirm the observations by Bryan et al., who reported no IgG detection in samples taken from patients during periods before the COVID pandemic using Architect Abbott´s SARS-CoV-2 IgG^16^. Furthermore, we show the same lack of cross-reactivity in the Panbio^TM^ COVID-19 IgG/IgM rapid test device. We chose patients with cured proven COVID-19 (Cohort 2) to study the performance of Architect Abbott´s SARS-CoV-2 IgG, but the rate of false-negative serologic results increased 25%. This rate was surprisingly high given that serum samples were taken at a median of 26 days from the onset of symptoms. This may indicate the existence of a group of outpatients for whom the role of serology testing is lower than for hospitalised and sicker subjects. Patients in Cohort 2 may be representative of the general population who passed COVID-19 and did not attend the hospital. We found that CLIA index values in serum samples taken ≥ 3 weeks after symptoms onset from patients in Cohort 1 were invariably high. Values in samples from patients in Cohort 2 were wider and some of them high as well. It should be noted that the number of samples taken from the second week of symptoms onset on in patients from Cohort 2 was higher compared to patients from Cohort 1, what could explain the differences.

Considering the data presented in this study, we conclude that IgG detection is insufficient to assess the current percentage of subjects with cured infections. Other authors have also failed to detect IgGs in 15%-20% of COVID-19-positive patients^7,9,11,14^. Sensitivity of IgGs antibody detection against either the SARS-CoV-2 nucleoprotein or the spike protein receptor-binding domain could make the difference^10^. Since Architect Abbott´s SARS-CoV-2 IgG detects anti-nucleoprotein antibodies, we cannot rule out the presence of antibodies against other viral epitopes.

We compared study patients grouped by IgG detection results aiming to discover potential predictors of IgG-negative patients with COVID-19 in both cohorts. We failed to find such predictors, which illustrates the difficulties to determine which patients will not have detectable IgGs. Whether the absence of detectable IgG antibodies in patients with cured infection means a lack of protection to prevent a new infection remains to be defined.

Our study is subjected to limitations. IgM detection using the Architect analyser was not available at the moment of the study. Moreover, it was not possible to collect additional serum samples from patients in Cohort 1 in order to check if false-negative determinations may have turned positive over time.

In conclusion, IgG detection alone is insufficient for the diagnosis of active and cured COVID-19. IgM has marginal diagnostic value. Coupled detection of SARS-CoV-2 RNA and IgG may improve the performance of any of the two procedures alone. The Panbio^TM^ COVID-19 IgG/IgM rapid test device is a good point-of-care alternative to Abbott´s SARS-CoV-2 IgG.

## Methods

### Hospital setting

The study was conducted at Gregorio Marañón hospital, a 1550-bed large tertiary hospital located in a centric area of Madrid, Spain. The COVID-19 epidemic had a great impact on this hospital during March and April 2020. The emergency department cared for a mean number of 133 patients with clinical suspicion of COVID-19 every day between March 15 and April 15, 2020.

### Study patients

Patients with clinical signs and symptoms of COVID-19 in the epidemic scenario were classified considering PCR results on nasopharyngeal samples and radiological findings as either having a proven (positive PCR result) or probable disease (negative PCR but a suggestive radiological pattern)^20^. Asymptomatic subjects by the time of sample collection were considered as not infected (if PCR proved to be negative) or asymptomatic carriers (if PCR proved to be positive). Three cohorts of patients were chosen to assess the role of antibody detection for the diagnosis of COVID-19. Cohort 1 (n = 65) included patients with clinical suspicion of active COVID-19 who attended the emergency department on March 30, 2020. Cohort 2 (n = 92) were hospital workers who had either been (n = 40) or not been (n = 52) diagnosed with proven/probable COVID-19 in previous months; all patients were asymptomatic at the time of the sampling. Paired nasopharyngeal swabs and venipuncture-obtained serum samples were collected from patients in Cohort 1 (March 30) and cohort 2 (April 3 to 22). Cohort 3 (n = 38) involved patients cared at the hospital in 2018 and 2019 and diagnosed with either community-acquired pneumonia of unknown etiology (n = 19) or a coronavirus infection other than SARS-CoV-2 (n = 19).

### Detection of viral RNA and antibodies against SARS-CoV-2

Nasopharyngeal samples were tested for the presence of viral RNA (the TaqManTM 2019-nCoV assay; Applied Biosystems, Pleasanton, CA, USA). Detection of serum IgG antibodies against the SARS-CoV-2 nucleocapsid protein was carried out in the Architect analyser using Abbott's SARS-CoV-2 IgG assay (Abbott, Abbott Park, IL, USA) following manufacturer´s instructions. The assay is based on a chemiluminescent microparticle immunoassay and determinations were considered negative or positive depending if results were < 1.4 or ≥ 1.4, respectively (cut-off index value). Detection of IgG and IgM antibodies was performed using the immunochromatographic Panbio^TM^ COVID-19 IgG/IgM rapid test device (Abbott, Abbott Rapid Diagnostic, Jena GmbH, Jena, Germany) following manufacturer´s instructions.

### Clinical data and analysis

The following demographic and clinical data were collected: age, sex, presence of symptoms compatible with COVID-19 (fever > 38 °C, headache, cough, asthenia, myalgia, sore throat, runny nose, dyspnoea, anosmia, dysgeusia, abdominal pain, and diarrhoea), date of symptom onset, hospital admission, radiological images, immunosuppression, and outcome (favourable or death during admission). Clinical data were collected in a pre-established protocol that was either retrospectively filled out retrieving the information from electronic charts for Cohort 1 patients or filled out at sample collection for Cohort 2 patients. Categorical variables were described and compared using Chi-square or Fisher´s exact tests and continuous variables were compared using the t-student and the Mann–Whitney U tests (IBM SPSS Statistics, version 26; Armonk, NY, USA).

Samples from patients in Cohort 3 were used to rule out antibody detection cross-reactivity. Sensitivity, specificity, positive predictive value, and negative predictive value were calculated separately for Cohorts 1 and 2. Independent analyses were carried out considering or excluding probable cases at different stages of the disease (less or more than seven days from clinical disease onset) for patients in Cohort 1; no further analyses of diagnostic performances were done for subjects in Cohort 2 given that only one patient had probable COVID-19 and all samples were taken > 7 days after the onset of the symptoms. We compared the results generated by Abbott´s SARS-CoV-2 IgG assay and the Panbio^TM^ COVID-19 IgG/IgM rapid test device. Representation of index values of IgG´s detected by Abbott´s SARS-CoV-2 IgG assay was done using scatter plots (Graph Pad Prism 5.02 statistical software; GraphPad, La Jolla, CA, USA).

### Ethical considerations

This study was approved by the Ethics Committee of Hospital Gregorio Marañón (CEIm; studies no. MICRO.HGUGM.2020–023 and MICRO.HGUGM.2020–021). All research was performed in accordance with relevant guidelines/regulations. Informed consent was obtained from patients in Cohort 2; a waiver for informed consent from patients in Cohort 1 and Cohort 3 was obtained given the retrospective designed of the samples collection from those patients.
